# Appendiceal Endometriosis and Carcinoid Presented as Acute Appendicitis in Pregnancy: A Rare Case Report and Review of the Literature

**DOI:** 10.1155/2013/360459

**Published:** 2013-02-27

**Authors:** Panagiotis A. Dimitriadis, Ragai R. Makar, Gearoid Kingston, Ridzuan Farouk

**Affiliations:** ^1^General Surgery Department, Royal Berkshire Hospital, London Road, Reading RG1 5AN, UK; ^2^Pathology Department, Royal Berkshire Hospital, Reading RG1 5AN, UK

## Abstract

A 22-year-old pregnant woman presented at the twenty-seventh week of gestation in the Emergency Department with acute abdominal pain and right iliac fossa tenderness. Urgent MRI was done and was suggestive of acute appendicitis. A laparoscopy was performed that confirmed an inflamed and purulent appendix that was removed. The technique used is described in detail. The histopathologic findings were those of acute appendicitis, carcinoid, and endometriosis of the appendix. We report the first case of this extremely rare triad presented in pregnancy.

## 1. Introduction

Acute appendicitis is the most common surgical emergency encountered by the general surgeon. Its incidence in pregnancy has been found to be less than 1 in 1500 [1]. Appendiceal endometriosis and carcinoid presented as acute appendicitis in pregnancy are extremely rare; to our knowledge this is the first case reported in the literature. We managed this emergency laparoscopically; the technique used is described in detail. Laparoscopy in pregnancy is safe and reduces length of stay and postoperative pain [2]. 

## 2. Main Text

A 22-year-old pregnant woman, gravida 2, para 1, presented at the twenty-seventh week of gestation in the Emergency Department with a 6-hour history of right-side abdominal pain. The pain started at rest and was stabbing in nature. It was worse with movements and better with simple analgesia. She was anorexic but had no nausea or vomiting. There was no history of vaginal bleeding or dysuria. Past medical and surgical histories were unremarkable. On examination, the patient was lying still and pale. She was apyrexial with a heart rate of 80 beats per minute, blood pressure of 110/60, and O_2_ saturations of 97% on air. Her chest was clear, and her abdomen was tender at the right iliac fossa with rebound tenderness but no guarding. Rovsing's sign was positive, and the bowel sounds were present and normal in nature. Vaginal examination was negative, and there was no uterine tenderness. Laboratory studies showed a white blood cell count of 13.14 × 10^9^ g/L (normal value 4.0–10.50 × 10^9^ g/L), neutrophils 9.20 × 10^9^ g/L (normal value 1.80–7.50 × 10^9^ g/L) and C-reactive protein level of 15.1 mg/L (normal value 0–4.9 mg/L). 

**Figure 1:**
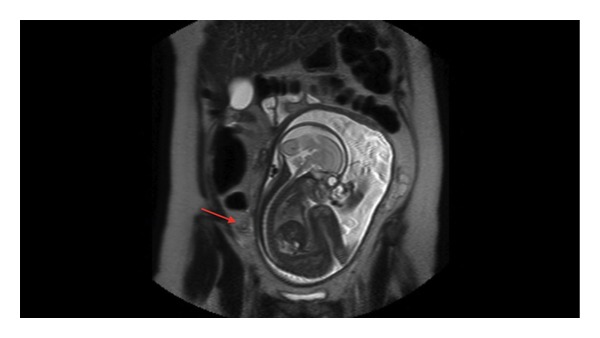
MRI T2 coronal view: free fluid and inflammatory changes around the appendix (arrow). Gravid uterus.

The patient's clinical presentation was highly suspicious of an acute appendicitis. An urgent (Magnetic Resonance Imaging) MRI scan was done to eliminate other differential diagnosis. This scan showed mild periappendiceal fat stranding, suggestive of early acute appendicitis (Figure 1). 

The patient underwent laparoscopy under general anesthesia. The fetus was monitored perioperatively. The first port was introduced via the left upper quadrant using Visiport 12 mm, which was followed by the 5 mm post at the right upper quadrant and then 12 mm post at the suprapubic midline under vision. We used pneumoperitoneum pressure of less than 14 mmHg. 

A small amount of free fluid was encountered in the right lower abdomen. After identification of the ileocecal region, an inflamed and nonperforated purulent appendix was found. The uterus was gravid, and the fallopian tubes and ovaries were normal. The mesoappendix was divided with diathermy to a healthy base. The latter was secured with PDS (polydioxanone) endoloop, and the appendix was removed in a retrieval bag via the suprapubic port. 

Thorough irrigation of the lower abdomen with normal saline was followed. The 12 mm port sites were closed with PDS 0 sutures. The patient had uneventful postoperative recovery. She was discharged home at the third postoperative day. Three months later, she had normal vaginal delivery of healthy full-term child.

The entire specimen has been examined histologically. The appendix showed luminal pus, patchy mucosal ulceration, transmural acute inflammation, and overlying serositis, in keeping with an acute appendicitis.

**Figure 2:**
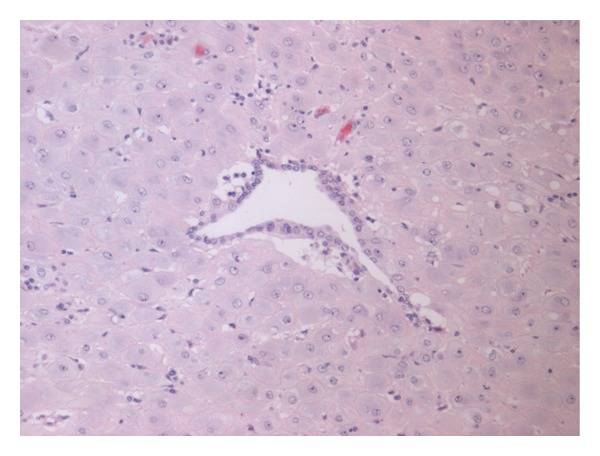
Endometriosis H&E ×100. A central endometrial gland surrounded by abundant decidualised endometrial stroma.

There was an abundant decidualised endometriosis (Figure 2) within the wall of the appendix, as confirmed by CK7 and CD10 immunohistochemistry. The endometriosis is likely to be longstanding, but the endometrial stromal decidualisation is presumably a response to marked elevation of systemic progesterone in pregnancy. The considerable and rapid expansile effect of endometrial stromal decidualisation is presumably responsible for the acute appendicitis, which led to the clinical presentation.

**Figure 3:**
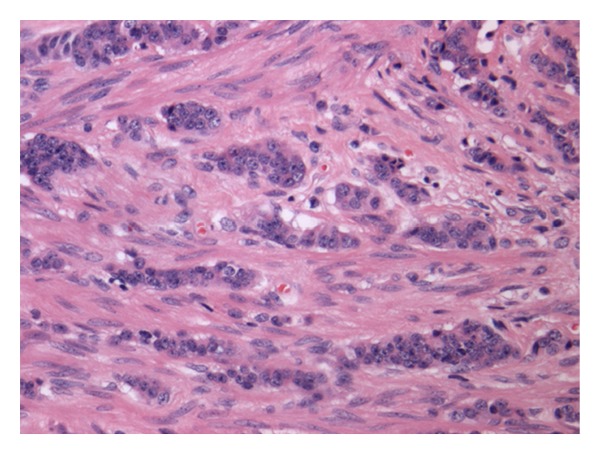
Well-differentiated endocrine tumour (classic carcinoid) H&E ×200 Nests and cords of uniform, bland, cohesive tumour cells with round nuclei, stippled chromatin, and eosinophilic cytoplasm.

In addition, at the tip of the appendix, there was a well-differentiated endocrine tumour (Figure 3) (G1, classic carcinoid) measuring 4.5 mm in maximum dimension, which was largely confined to the muscularis propria, but which very focally invaded (0.2 mm) subserosal tissues, in keeping with stage pT2 tumour. Nests and cords of uniform, bland, cohesive tumour cells with round nuclei, stippled chromatin, and eosinophilic cytoplasm were seen. Endocrine differentiation has been confirmed by immunoexpression with synaptophysin, chromogranin A, and CD56. A MIB1 proliferation index of less than 2% confirmed that this is a grade 1 tumour. Given its small size, the tumour was presumed to be an incidental finding.

## 3. Discussion

The simultaneous presentation of acute appendicitis, endometriosis, and carcinoid tumour of the appendix during pregnancy is extremely rare; to our knowledge, this is the first case described in the literature.

 Although acute appendicitis is the most common surgical emergency encountered by the general surgeon, its incidence in pregnancy has been found to be less than 1 in 1500 [1].

Laparoscopy in pregnancy has become a more common and accepted surgical approach; however some clinicians still have concerns about the safety of pneumoperitoneum for the fetus and the mother. Large studies that compared laparoscopy and laparotomy for nonobstetric procedures found similar outcomes. Moreover, the length of stay, postoperative pain, and infection rates are generally lower in laparoscopy [2]. We used the Visiport through the left upper quadrant instead of umbilical port, because of its advantage in avoiding the potential difficulty because of the gravid uterus and also to minimize the risk of uterine injury. This technique allowed us the proper planning of the other two ports and to insert them under direct vision. 

Endometriosis is defined as the presence of endometrial tissue outside the uterine lining and is a fairly common disease. The appendix is rarely implicated: 2.8–4.1% of all cases of endometriosis and 4/1,000 of the general population [3, 4]. Most patients remain asymptomatic or present with acute appendicitis [5]. Acute appendicitis and appendiceal endometriosis complicate pregnancy with a frequency of 3–8/10,000 deliveries [6]. All patients found to have appendiceal endometriosis should be followed up postoperatively; treatment of any extra-intestinal deposits should be considered [7].

Carcinoid tumour is the commonest malignant neoplasm of the appendix. Others include: papillary mucinous cystadenocarcinoma, and nonpapillary adenocarcinoma [8]. It was found in 2.7/1,000 appendicectomy specimens [1] and comprises almost 19% of all carcinoid tumours in the body [9]. As with endometriosis, carcinoid of the appendix either remains asymptomatic or presents as acute appendicitis [10]. A review of the literature reveals 20 cases of carcinoid of the appendix during pregnancy in the last 67 years [11, 12]. Carcinoids are usually found at the tip of the appendix; those less than 1 cm in diameter are virtually nonmetastatic and are treated with simple appendicectomy. For those more than 2 cm, there is an increased risk of regional spread and distant metastasis and a right hemicolectomy is recommended. A postoperative follow-up is recommended as there is a high risk of synchronous and metachronous colorectal cancer (13–33%) [12]. With regards to the pathophysiology of acute appendicitis in the context of appendiceal endometriosis and carcinoid tumour, the data is limited. It seems that there is a degree of luminal obstruction, which increases the appendiceal luminal pressure distally; vascular supply is compromised and this initiates the acute inflammatory response seen in cases of appendicitis.

## 4. Conclusion

To our knowledge, this is the first report of the simultaneous occurrence of appendiceal carcinoid tumour and endometriosis presenting as acute appendicitis during pregnancy. Early surgical intervention of these patients will prevent complications such as perforation and peritonitis, which are potentially fatal for both the mother and the fetus. Laparoscopy enables the examination of the peritoneal cavity providing an excellent diagnostic and therapeutic procedure with minimal postoperative complications compared to the open surgery. Moreover, it can be safely performed during pregnancy. Finally, the definitive diagnosis of appendiceal endometriosis/carcinoid will be made under the microscope. We need to consider such conditions that may mimic or present with acute appendicitis and follow the patients up postoperatively as required.
